# Case Report: Indwelling Pleural Catheter Based Management of Refractory Hepatic Hydrothorax as a Bridge to Liver Transplantation

**DOI:** 10.3389/fmed.2021.695977

**Published:** 2021-07-12

**Authors:** Mayurun Selvan, Hannah Collins, William Griffiths, William Gelson, Jurgen Herre

**Affiliations:** ^1^Respiratory Medicine, Cambridge University Hospitals National Health Service Foundation Trust, Cambridge, United Kingdom; ^2^Cambridge Liver Unit, Cambridge University Hospitals National Health Service Foundation Trust, Cambridge, United Kingdom

**Keywords:** indwelling pleural catheter, hepatic hydrothorax, liver transplantation, pleural disease, case series

## Abstract

**Introduction:** Liver transplantation is the treatment of choice for decompensated liver disease, and by extension for hepatic hydrothorax. Persistent pleural effusions make it challenging for patients to maintain physiological fitness for transplantation. Indwelling pleural catheters (IPCs) provide controlled pleural fluid removal, including peri-operatively. The immune dysfunction of cirrhosis heightens susceptibility to bacterial infection and concerns exist regarding the sepsis potential from a tunnelled drain.

**Method:** Six patients were identified who underwent IPC insertion for hepatic hydrothorax before successful liver transplantation, between November 2016 and November 2017.

**Results:** All patients had recurrent transudative right sided pleural effusions. Mean age was 49 years (range 24–64) and mean United Kingdom Model for End-Stage Liver Disease score was 58. Four patients required correction of coagulopathy before insertion. There were no complications secondary to bleeding. Three patients were taught self-drainage at home of up to 1 litre (L) daily. A protocol was developed to ensure weekly review, pleural fluid culture and drainage of larger volumes in hospital. For every 2–3 L of pleural fluid drained, 100 mls of 20% Human Albumin Solution (HAS) was administered. On average an IPC was *in situ* for 58 days before surgery and drained 19 L of fluid in hospital. There was a small increase in average BMI (0.2) and serum albumin (2.1 g/L) at transplantation. There was one episode of stage one acute kidney injury secondary to high volume drainage. No further ascitic or pleural procedures were needed while an IPC was *in situ*. One thoracentesis was required after IPC removal. On average IPCs remained *in situ* for 7 days post transplantation and drained a further 2 L of fluid. Pleural fluid sampling was acquired on 92% of drainages in hospital. Of 44 fluid cultures, 2 cultured bacteria. Two patients had their IPCs and all other lines removed post transplantation due to suspected infection.

**Conclusion:** Our case series describes a novel protocol and successful use of IPCs in the management of refractory hepatic hydrothorax as a bridge to liver transplantation. The protocol includes albumin replacement during pleural drainage, regular clinical review and culture of pleural fluid, with the option of self-drainage at home.

## Introduction

Hepatic hydrothorax is a pleural effusion in patients with cirrhotic liver disease, without cardiopulmonary disease (1). It is a complication in 5–10% of patients with cirrhosis (1–3), and associated with significant morbidity (1, 4) and mortality (5). The pathophysiology is believed to involve the direct movement of ascitic fluid into the pleural cavity through diaphragmatic defects, due to negative pressure within the pleural space (4, 6, 7).

Medical management typically involves a low salt diet and diuretics, with therapeutic thoracentesis as required. Up to 26% of hepatic hydrothoraxes will be refractory to treatment and cause persistent symptoms such as cough and breathlessness (4, 7). Management of refractory hepatic hydrothoraces has historically proven to be difficult. Liver transplantation is the treatment of choice for decompensated liver disease (8), and by extension is the ideal management of hepatic hydrothorax (9). This option is rarely immediately available (10). Large, persistent pleural effusions make it challenging for patients to maintain the physiological fitness required whilst awaiting transplantation.

There has been increasing discussion regarding management of hepatic hydrothorax in the bridging period to transplantation. The aim is alleviation of dyspnoea with removal of pleural fluid, however any intervention must be carefully weighed up against the risks which could jeopardise a future lifesaving treatment.

Indwelling pleural catheters (IPCs), known to be effective in management of malignant effusions, have been increasingly used in management of benign pleural effusions (11). IPCs are an attractive prospect in the management of hepatic hydrothorax as they provide controlled pleural fluid removal including in the peri-operative setting. Given the immune dysfunction of cirrhosis, coupled with increased translocation in portal hypertension, heightens susceptibility to bacterial infection (12), concerns exist regarding the sepsis potential from a tunnelled drain, that could preclude transplantation.

This case series describes six patients who underwent IPC insertion to manage hepatic hydrothoraces, followed by definitive liver transplantation.

## Method

Six patients were identified who underwent IPC insertion for hepatic hydrothorax between November 2016 and November 2017 at Cambridge University Hospital. All patients went on to have successful liver transplantation. Information regarding patient demographics, aetiology of liver disease, amount and frequency of drainage, and serial microbiology and blood tests results were obtained from the patient's online notes, Epic® (*Epic Systems Corporation)*. Verbal and written consent has been obtained from all six patients.

## Results

**Figure 1:**
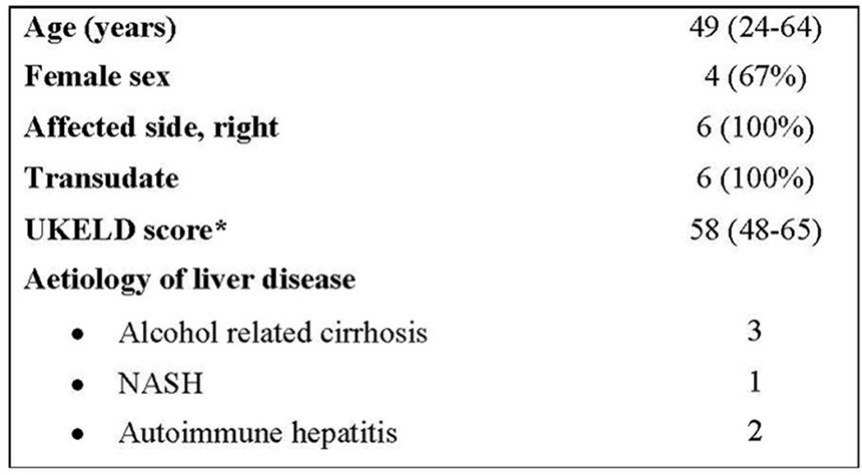
Demographics. *A United kingdom Model for End-Stage Liver Disease (UKELD) score of 49 indicates a 9% 1-year risk of mortality, and a score of 60 indicates a 50% chance of 1-year survival (13).

Demographics for our six patients are shown in Figure 1.

All six patients were inpatients under the care of the hepatology team at the point of referral for IPC insertion and suffered from recurrent right-sided transudative pleural effusions. All had been medically managed with diuretics and had at least one pleural procedure previously. None of the patients had previously had transjugular intrahepatic portosystemic shunt procedures. Their average United Kingdom Model for End-Stage Liver Disease (UKELD) score was 58, suggesting a 1 year mortality of between 9 and 50%.

All patients had a 16F tunnelled IPC inserted by the interventional pleural team, under ultrasound guidance. Clotting screen and platelet counts were checked in all patients pre-procedure. At insertion the average international normalised ratio (INR) was 1.4, and the average platelet count was 133 ×10^9^/L.

Four patients had blood products given to correct coagulopathies before the procedure was undertaken. All four patients had fresh frozen plasma, two had platelet transfusions and one was given intravenous vitamin K. During the procedure all patients were given 15–20 mls of 2% local anaesthetic with adrenaline (1:200,000) and there were no complications secondary to bleeding.

Suitable patients (patients 4, 5 and 6 in Table 1) were trained to use drainage kits at home to allow a maximum of 1 L daily drainage, with weekly review by the pleural team to drain larger volumes (to dryness) with albumin replacement. Patient 6 had their follow-up reviews at their local hospital. Home drainage was initiated immediately the day after insertion as these patients had prior large volume aspirations without evidence of renal compromise. Those patients who drained their IPC at home reported daily drainage of close to 1 L to their clinicians. The volume of fluid drained at home is included in the data in Table 1, though the total amount drained is based on an estimate as there were no complete formal records of home drainage.

**Table 1:** Results.

**Patient**	**1**	**2**	**3**	**4**	**5**	**6**	**Mean**
**Before insertion of drain**							
Ascitic drains in 12 month prior	3	0	>3	1	2	0	
Previous pleural procedures	3	1	2	1	2	4	2.2
**Between insertion and transplant**
Insertion to transplant (days)	78	16	3	82	135	31	57.5
Total drain time *in situ* (days)	85	21	5	92	140	46	64.8
BMI change	0.9 (+)	0	0	1.1 (+)	0.4 (+)	1.0 (–)	0.2 (+)
Serum Albumin change	7 (–)	9 (+)	8 (+)	2 (–)	1 (+)	4 (+)	2.1 (+)
Pre-transplant drain output in hospital (L)	35	14.6	5.3	26	31.2	1.5	18.9
Total estimated pre-transplant drain output total including home drainage (L)	35	14.6	5.3	97	146.2	31	54.8
Total times drained in hospital	9	6	1	11	20	1	8.0
Total times drained (estimated) including home drainage	9	6	1	82	135	31	51.8
Average output in hospital per drainage (L)	3.9	2.4	5.3	2.4	1.6	1.5	2.9
Average output per drainage (based on total estimated)	3.9	2.4	5.3	1.2	1.1	1	2.5
20% HAS requirements (L)	1.2	1.4	0.3	1.2	2.6	0.4	1.2
Fluid cultures sampled	9	2	1	10	20	2	7.3
**Post transplantation**							
Days drain *in situ* post-transplant	6	5	2	10	5	15	7.2
Post transplantation drain output (L)	1.7	2	0.4	1	3.9	3.4	2.1

A local protocol, based on our institution's peritoneal drainage protocol, was developed in conjunction with our Hepatology department to ensure regular in person review, regular culturing of pleural fluid and supervised drainage of larger volumes of fluid in hospital (for both inpatients and outpatients). For every 2–3 litres of pleural fluid drained, 100 mls of 20% Human Albumin Solution (HAS) was administered, with a view to reduce the risk of circulatory dysfunction and renal impairment. The volume of albumin administered was calculated based on the total drainage for the week, including drainage at home and on the day of review in hospital.

**Figure 2:**
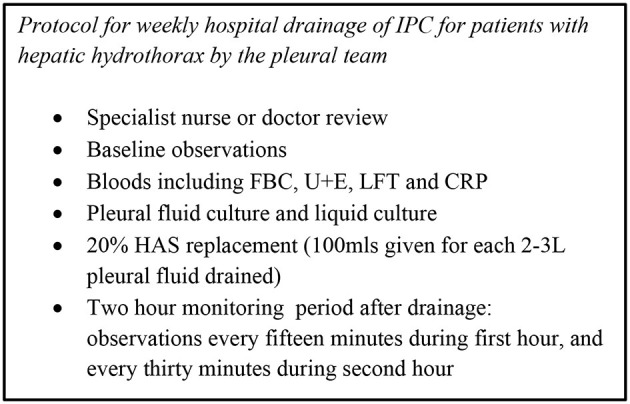
Protocol.

This protocol is illustrated in Figure 2.

On average an IPC was *in situ* for 58 days before surgery, with an average drainage of 19 litres of fluid in hospital in this time period. Pleural fluid culture sampling was acquired on 92% of drainages in hospital. Of the 44 fluid pleural fluid cultures sent on our series, two cultured bacteria – *Acinetobacter Iwoffi* (a known skin coloniser) and *Streptococcus parasanguinis*.

The IPCs remained *in situ* for an average of 7 days post liver transplantation and on average drained 2 litres of pleural fluid after surgery. One patient required a single further thoracentesis post IPC removal. No patients required further ascitic or pleural drainage while the IPC was *in situ*.

Our patients on average had a small increase in albumin and BMI between insertion of the IPC and liver transplantation. A single patient had a single episode of stage I AKI associated with high volume pleural drainage, as described above, despite human albumin replacement. There was no significant derangement in sodium or creatinine associated with pleural drainage seen otherwise. All patients remain under ongoing follow up by the Hepatology team at our institution.

Table 1 summarises the individual data collected from our six patients.

One patient was noted to have a pneumothorax on their post IPC insertion chest x ray – to manage this the IPC was connected to an underwater seal and not clamped. As a result, pleural fluid drainage was not controlled. Though 20% HAS was given, this resulted in acute kidney injury (AKI) stage 1, which resolved within a few days. The pneumothorax resolved within 24 h, and was likely related to high volume drainage.

There were no complications secondary to infection in our series prior to transplantation. One pleural drain was removed (in addition to all other lines and drains) in a patient after transplantation due to pyrexia of unknown source. *Streptococcus parasanguinis* was cultured post transplantation from pleural fluid on one occasion. This patient subsequently required one further thoracentesis after IPC removal. Another patient had their pleural drain removed on day 15 after transplant as a precaution due to a suspected infection of unknown source. No organisms were cultured in pleural fluid, sampled at the time of removal of the IPC.

## Patient Perspective

All our patients reported breathlessness as a symptom (to varying degrees) and reported an improvement in breathlessness with drainage of pleural fluid. One of our patients had a limited memory of that time period, and had their IPC *in situ* for a short period of time.

The remaining patients stated a preference for IPC management of their pleural effusions, compared to repeated thoracentesis. Their comments included that repeat therapeutic aspirations tended to be time consuming and uncomfortable, and performed on an emergency basis.

Our patients spoke favourably of having weekly clinical review, and did not mind the distance required to travel to hospital. One patient described feeling more secure knowing that they would have regular scheduled review, and all patients spoke positively of the relationship that was built due to the continuity of care.

Three of our patients performed regular drainages away from the hospital – one at their local surgery, and two at home, with their spouses performing drainage. All three spoke favourably of home drainage, citing increased autonomy and having more control of their own symptoms. They reported no technical issues with draining their IPCs, and all had a point of contact for any concerns.

One patient reported mild discomfort at the site following insertion. Another, who had been taught to access the IPCs while they were in hospital, described that they had less confidence in ward nurses unfamiliar with IPCs.

Our patients expressed positive sentiment towards IPC management of their effusions, with all patients stating they would opt for a tunnelled drain should they have to go through a similar presentation again. In particular, the autonomy and comfort of home drainage (or the potential of it) was mentioned as a positive, as was the comparison with experiences of recurrent aspirations prior to drain insertion.

## Discussion

We report a case series of six patients who successfully underwent IPC insertion for refractory hepatic hydrothorax as a bridge to liver transplant. We believe this is the largest case series in the UK. This remains an ongoing approach, with three further patients managed since our case series, all undergoing IPC insertion as bridging to successful liver transplantation.

Management of hepatic hydrothorax is centred around alleviating dyspnoea by control or removal of pleural fluid which can rapidly reaccumulate. Bhatnagar et al. analysed the data from 57 patients who underwent IPC insertion for benign pleural effusions, including 19 patients with hepatic hydrothorax who were not listed for transplant and found that patients with hepatic hydrothorax had undergone more pleural procedures before IPC insertion (median 4.5) with higher volumes of average weekly drainage (5.14 L per week) (11). Our patients drained an even higher estimated volume of 6.79 L per week.

Chest tube insertion in this context has been shown to carry increased morbidity and mortality with complications including renal dysfunction (14, 15). High volume drainage of fluid and re-accumulation of pleural fluid on clamping intercostal drains is characteristic. As a result, repeat thoracentesis has been the routine procedure for removal of pleural fluid from the pleural cavity and has been shown to have reduced mortality and hospital stay compared to chest drain insertion (16). However, a single centre retrospective review of patients undergoing thoracentesis showed the cumulative risk of complications increased with sequential thoracenteses (17), with increased risk of haemothorax with thrombocytopenia and higher MELD scores (Model for End-Stage Liver Disease which stratifies severity of liver disease). Repeated thoracentesis has also been shown to be associated with increased hospital cost and longer length of stay (18).

TIPSS (transjugular intrahepatic portosystemic shunt) procedures have been shown to be effective in the treatment of refractory hepatic hydrothorax. In a study of 24 consecutive patients with cirrhosis there was improvement in symptoms in 79% of patients. However, complete relief of symptoms was found to occur in only 58% of patients, with a further 21% of patients requiring further thoracentesis post procedure (19). A systematic review and meta-analysis found similar figures, with a complete response in 55% of patients, partial response in 18% of patients and an absent response in 21% of patients (20).

As IPCs are being more commonly used in the management of benign effusions, there is increasing work looking at a possible role in treating hepatic hydrothoraces, including bridging to transplantation and as palliation. A single-centre prospective feasibility study of 25 prospective liver transplant candidates who underwent IPC insertion for hepatic hydrothorax included five patients who went on to have liver transplantation (21). A retrospective review of sixty-two patients over a 10 year period who underwent IPC insertion for hepatic hydrothorax at a single referral centre, included ten patients who were bridged successfully to liver transplantation (22). The only multicentre retrospective review of use of IPC in hepatic hydrothorax included four patients who underwent liver transplantation (23).

In theory the use of an IPC to allow bridging to transplant is an attractive option. It allows controlled pleural fluid drainage in an inpatient or outpatient setting, and also allows the possibility of patients being able to access the drain at home for regular drainage. Given the large volumes of fluid that were drained in our series, this reduces emergency admissions and length of hospital stay due to breathlessness and importantly gives patients increased autonomy. In our case series no further pleural nor ascitic procedures were required while the IPCs were *in situ*.

IPCs also provide the possibility for controlled drainage peri-operatively, without need for further procedures. This was evident in our case series, with two patients draining in excess of 3 litres of fluid post-operatively before IPC removal. Post-operative chest drainage was explored in a retrospective study of 597 patients who underwent liver transplantation in which as many as 361 (60%) patients required a chest drain within the first 10 days of surgery (24).

Spontaneous pleurodesis did not occur before liver transplantation in any of our patients. Chen et al. found a spontaneous pleurodesis rate of 33% in their prospective study of IPC in hepatic hydrothorax with an average time to pleurodesis of 132 days (21), a longer period on average than patients in our series had their IPC *in situ* before transplantation. Bhatnagar et al. found a median pleurodesis time of 222 days and lower pleurodesis rate (11%) in hepatic hydrothorax compared to effusions due to other pathologies and suggested IPC use as a long-term symptomatic palliative measure or as a bridge to transplantation (11).

Two of our patients had their IPCs removed post-operatively, in addition to all other lines, due to suspected infection. This highlights the concerns regarding pleural infection associated with long term indwelling pleural catheters. In the multicentre retrospective study by Shojaee et al. of 79 patients who had IPC inserted due to hepatic hydrothorax, they found a 10% overall infection rate and 2.5% mortality rate because of IPC-related empyema and sepsis. Of particular concern, IPC prevented liver transplant in one patient because of IPC-related sepsis and death (23). In the retrospective study of 62 patients by Kniese et al. two patients who were listed for transplant died secondary to IPC-related empyema. This study described an 18% incidence of pleural infection (22). The single centre prospective feasibility study by Chen et al. found pleural infection rates of 16%, however with no associated mortality (21).

In a recent editorial, Walker and Maldonado describe the difficulties of characterising pleural infection in hepatic hydrothorax, in particular due to the rate of spontaneous bacterial empyema (25) (13%) and colonisation of the IPC that can be difficult to distinguish from genuine pleural infection. Despite this, the rates of infection and mortality described in a cohort of patients awaiting a potentially lifesaving procedure necessitate high levels of vigilance. As a result, they advise that IPCs “should be considered with great caution in patients eligible for liver transplantation” (26).

We specifically developed a local protocol (see Figure 2) as a safety mechanism to be able to respond to potential infection promptly. It ensured weekly clinical review by the pleural team, culture of pleural fluid in the majority of drainages in hospital and regular monitoring of inflammatory markers. This was particularly important in our series, which involved patients accessing the drain at home (after thorough training). There were no complications secondary to infection in our series prior to transplantation.

The retrospective study by Kniese et al. showed a small downward trend in albumin and BMI in their cohort of patients (22). It is unclear if replacement of pleural fluid with HAS, might reduce nutritional or electrolyte derangement (26). HAS is used routinely in large volume paracentesis and recommended by the American Association for the Study of Liver Diseases (27) and the European Association for Study of the Liver (28). A systematic review and meta-analysis suggested that “use of albumin in paracentesis was associated with significantly reduced risk of paracentesis-induced circulatory dysfunction (OR 0.26 95%, CI 0.08–0.93) and there was a non-significant difference in death, encephalopathy, hyponatraemia, readmission and renal impairment” (29). In our series patients were given 100 mls of 20% HAS for every 2–3 litres of pleural fluid drained, as per the agreed protocol with our Hepatology department. Apart from a single episode of stage 1 acute kidney injury, there was no significant derangement in sodium or creatinine associated with pleural drainage seen.

There are several limitations to our study. A dyspnoea assessment was not used to assess for any improvements in quality of life, though an improvement in breathlessness was described by all patients. Selection bias is likely as this is a small case series of patients who were selected as they successfully underwent liver transplantation. Additionally, for three of our patients the total volume of fluid drained during our study was estimated based on their verbal report, as there was no complete formal record of the daily volume drained by patients at home.

## Conclusion

Our case series offers a description of successful use of IPCs as management of refractory hepatic hydrothorax as a bridge to liver transplantation, using a novel protocol in which albumin replacement was given during pleural drainage, with weekly clinical review, regular culture of pleural fluid and monitoring of electrolytes, renal function and inflammatory markers. Larger retrospective studies have raised concerns regarding the potential for infection precluding liver transplantation, but have suggested a role for IPCs in palliation. A randomised controlled trial assessing the use of IPC's, as well as the role of albumin replacement, in hepatic hydrothorax as a bridge to transplantation, using a standardised management and monitoring protocol, is required.

## Data Availability Statement

The raw data supporting the conclusions of this article will be made available by the authors, without undue reservation.

## Ethics Statement

Written informed consent was obtained from the individual(s) for the publication of any potentially identifiable images or data included in this article.

## Author Contributions

MS collected and analysed data and drafted the manuscript. HC helped to identify patients to be included, was involved in the care of the patients and reviewed the manuscript. WGr and WGe reviewed and revised the manuscript. JH conceptualised the manuscript and identified the patients, revised the manuscript and provided final approval. All authors contributed to the article and approved the submitted version.

## Conflict of Interest

The authors declare that the research was conducted in the absence of any commercial or financial relationships that could be construed as a potential conflict of interest.

## Abbreviations

AKI: acute kidney injury
HAS: Human Albumin Solution
IPC: indwelling pleural catheter
TIPSS: transjugular intrahepatic portosystemic shunt
UKELD: United Kingdom Model for End-Stage Liver Disease.
